# Mitochondrial Oxidative Phosphorylation Compensation May Preserve Vision in Patients with OPA1-Linked Autosomal Dominant Optic Atrophy

**DOI:** 10.1371/journal.pone.0021347

**Published:** 2011-06-22

**Authors:** Nicole J. Van Bergen, Jonathan G. Crowston, Lisa S. Kearns, Sandra E. Staffieri, Alex W. Hewitt, Amy C. Cohn, David A. Mackey, Ian A. Trounce

**Affiliations:** 1 Centre for Eye Research Australia, University of Melbourne, Royal Victorian Eye and Ear Hospital, Melbourne, Victoria, Australia; 2 University of Western Australia, Perth, Western Australia, Australia; 3 Lions Eye Institute, Perth, Western Australia, Australia; University of Medicine and Dentistry of New Jersey, United States of America

## Abstract

Autosomal Dominant Optic Atrophy (ADOA) is the most common inherited optic atrophy where vision impairment results from specific loss of retinal ganglion cells of the optic nerve. Around 60% of ADOA cases are linked to mutations in the *OPA1* gene. OPA1 is a fission-fusion protein involved in mitochondrial inner membrane remodelling. ADOA presents with marked variation in clinical phenotype and varying degrees of vision loss, even among siblings carrying identical mutations in OPA1. To determine whether the degree of vision loss is associated with the level of mitochondrial impairment, we examined mitochondrial function in lymphoblast cell lines obtained from six large Australian OPA1-linked ADOA pedigrees. Comparing patients with severe vision loss (visual acuity [VA]<6/36) and patients with relatively preserved vision (VA>6/9) a clear defect in mitochondrial ATP synthesis and reduced respiration rates were observed in patients with poor vision. In addition, oxidative phosphorylation (OXPHOS) enzymology in ADOA patients with normal vision revealed *increased* complex II+III activity and levels of complex IV protein. These data suggest that OPA1 deficiency impairs OXPHOS efficiency, but compensation through increases in the distal complexes of the respiratory chain may preserve mitochondrial ATP production in patients who maintain normal vision. Identification of genetic variants that enable this response may provide novel therapeutic insights into OXPHOS compensation for preventing vision loss in optic neuropathies.

## Introduction

Autosomal Dominant Optic Atrophy (ADOA, OMIM 165500), also known as Kjer's optic neuropathy [1] is the most common hereditary optic neuropathy with a prevalence at 1∶12000 [2]. ADOA leads to vision impairment due to the degeneration of retinal ganglion cells (RGCs) and their axons in the optic nerve [3], [4], [5]. The ADOA phenotype and degree of vision loss often varies considerably within a given pedigree, with a spectrum of vision loss ranging from mild to severe [6], [7]. The optic neuropathy often manifests in early childhood with reduced visual acuity, a predominantly blue-yellow dyschromatopsia and central scotoma (blind spot) [8], [9].

Over 60% of ADOA has been linked to mutations in the nuclear-encoded mitochondrial protein OPA1, with over 220 mutations identified to date [7], [10], [11], [12]. The OPA1 protein plays an important role in regulating mitochondrial inner membrane fusion [13], [14], [15], [16] and blocking the release of cytochrome c to prevent apoptosis [15]. A number of studies have demonstrated the role of OPA1 in maintaining an intact mitochondrial network through promoting mitochondrial fusion. This network allows the cell to rapidly respond to changing metabolic needs by adjusting mitochondrial distribution [17] and ensures adequate “mixing” of mitochondrial proteins and mitochondrial DNA (mtDNA). However it is becoming increasingly apparent that OPA1 also has a direct role in modulating cellular bioenergetic status. OPA1 has been shown to interact directly with OXPHOS complexes I, II and III but not IV [18]. Furthermore evidence of OXPHOS dysfunction and defective ATP production in OPA1 animal models [19], [20], [21], [22], [23], [24] and skeletal muscle from ADOA patients is accumulating [18], [25], [26].

The considerable inter- and intra-familial variation in visual acuity and penetrance within OPA1 pedigrees [1], [6], [27], [28], [29], [30], [31] has largely been ignored in prior studies of OXPHOS function in ADOA patient cohorts. These variations in phenotypes imply that aside from OPA1 mutations there may be other genetic, epigenetic and/or environmental modifiers influencing disease progression. The mitochondrial haplogroup has been excluded as a disease modifier in ADOA [32], [33]. There is currently no known cure or treatment for ADOA, and given the central role played by mitochondrial dysfunction in ADOA, mechanistic studies on mitochondrial function may reveal disease pathways amenable to therapeutic intervention.

Prior studies in LHON have demonstrated OXPHOS deficiency in lymphoblast mitochondria [34], [35]. We used a similar technique to demonstrate clear differences in OXPHOS function in lymphoblast mitochondria derived from a large cohort of ADOA patients with varying degrees of vision loss [7]. We report that lymphoblast mitochondria from ADOA patients with normal vision had increased respiration rates and increased complex II+III (linked) activity and complex IV protein levels compared to ADOA patients with poor vision. Further, the level of ATP synthesis in mitochondria of ADOA patients with near normal vision was similar to that of non-mutation carriers, but significantly higher than for ADOA patients with poor vision. These findings indicate that aspects of mitochondrial function segregate with visual acuity in ADOA patients, and point to potential therapeutic targets for preserving vision in this mitochondrial optic neuropathy.

## Results

### Pedigree and patient characteristics

**Figure 1:**
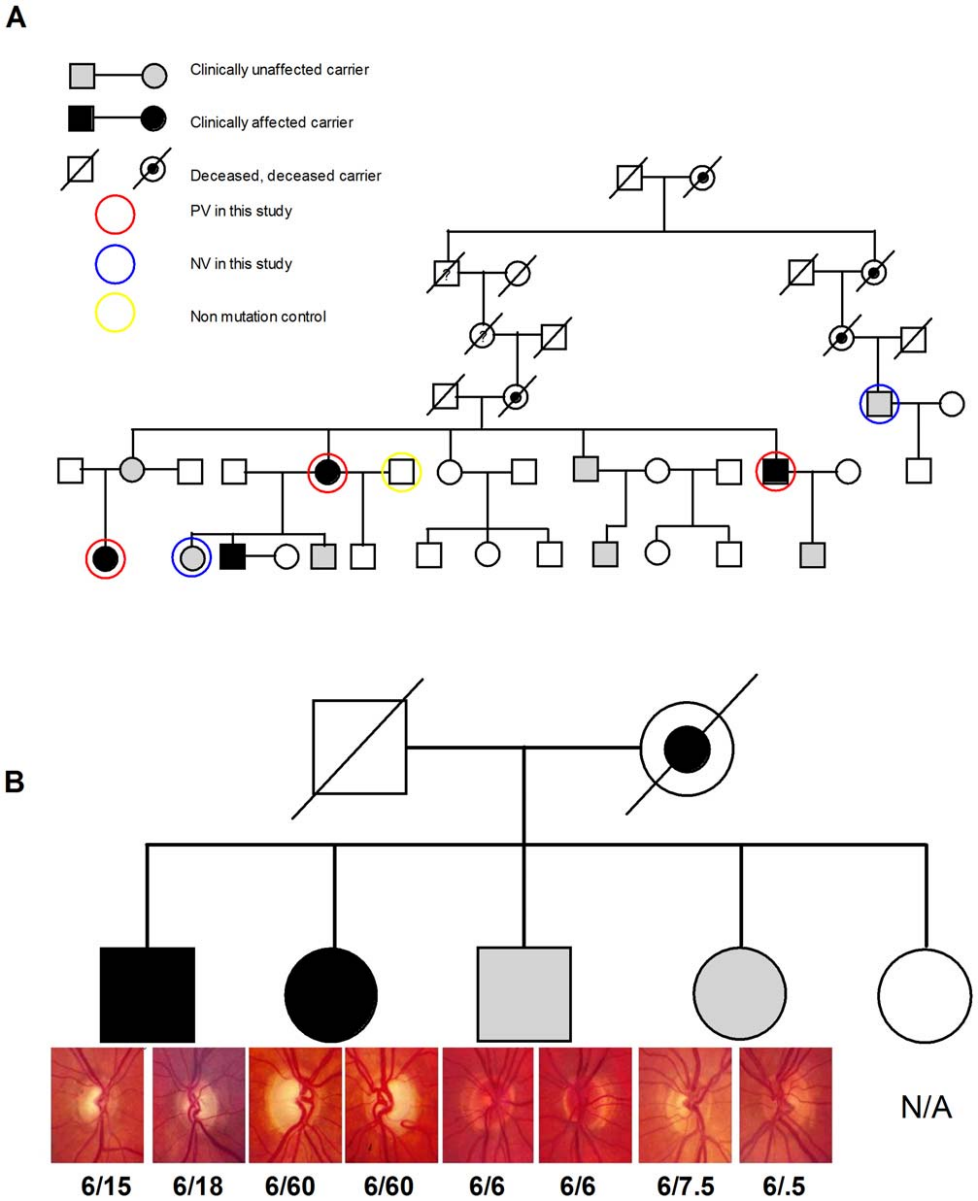
ADOA pedigree. A) A portion of the largest of the three pedigrees used in this study where patients are carrying the exon27 4bpdel 2708delTTAG OPA1 mutation. Black squares and circles indicate clinically affected, and white squares and circles indicate clinically unaffected members. The circled members were included in this study. B) An example of variation in fundus findings within a sibship is shown. Fundus photos from one sibship in the exon27 4bpdel 2708delTTAG OPA1 pedigree illustrate the highly variable clinical findings, showing thinning and pallor of the neuroretinal rim of the optic disc in the two patients at left compared with the two patients at right. Visual acuities are shown for each eye below the photographs. Variation in visual acuity was observed between siblings and parent-offspring.

**Figure 2:**
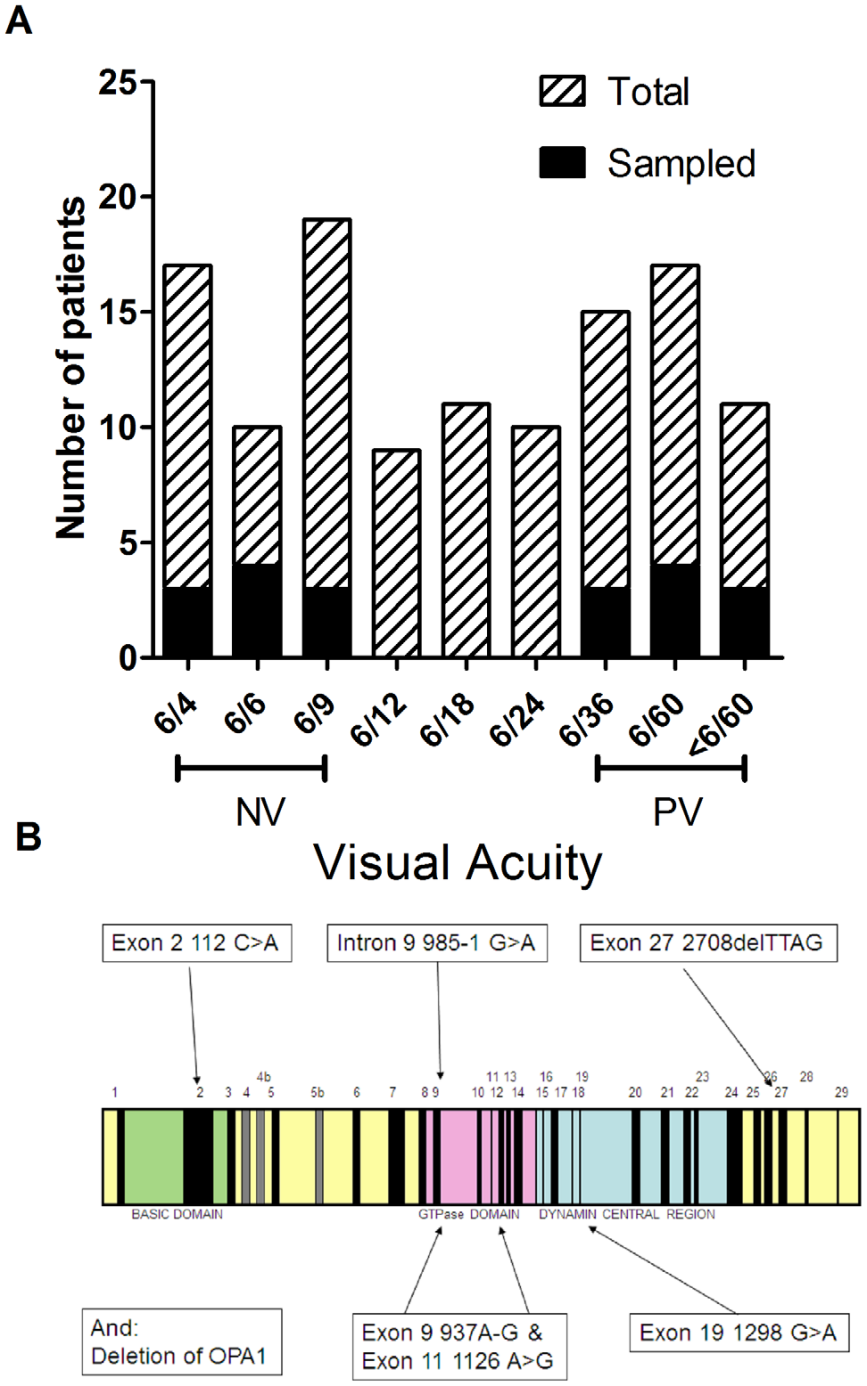
ADOA patient characteristics. A) Distribution of visual acuities (horizontal black bars) with mean visual acuity (white) per pedigree, and total visual acuity spread, and sampled patients (black) in our population. B) Map of the OPA1 gene and localisation of the mutations analysed in this study.

ADOA is characterised by varying degrees of vision loss in siblings with identical mutations in OPA1. An example family pedigree (Figure 1A) shows the clinical severity of ADOA in family members with the same exon27 4bpdel 2708delTTAG OPA1 mutation. The fundus photos from one sibship in the exon27 4bpdel 2708delTTAG pedigree illustrates the highly variable clinical findings between four siblings and parent-offspring (Figure 1B); the two siblings on the left with poor vision show thinning and pallor of the neuroretinal rim of the optic nerve head whilst the two siblings with near-normal vision have normal optic disc appearances. Visual acuities for each eye are shown below the photographs. There is a broad range of visual acuities observed within each pedigree as well as in the combined pedigrees (Figure 2A).

To identify potential mechanisms underlying the cause of vision loss in ADOA we sampled patients from six large Australian OPA-1 linked ADOA pedigrees. There are more than 220 known OPA1 mutations [12], and in this study each of these six pedigrees represented a different OPA1 mutation or substitution. We sampled patients from families that contained individuals in the highest and lowest tertile for visual acuity (Figure 2A). This selection included patients with either very poor vision (visual acuity 6/36 or less, n = 8) or with relatively normal vision (VA 6/9 or greater, n = 7). Across the six pedigrees there were a total of 158 patients carrying OPA1 mutations which have been extensively described [6], [7].

To select patients according to differing visual acuity levels, we selected families where siblings within the same family had a wide range of VA. This variation is well described in prior studies [1], [6], [27], [28], [29], [30], [31] and does not differ significantly according to the OPA1 mutation [6], [27]. Thus we believed that pooling of patients from different pedigrees containing various OPA1 mutations was justified as a means to examine biochemical changes associated with vision loss. Selected patients had stable vision. We have recently reported that overall within the wider cohort, visual acuities were stable in the majority of patients (72%) over a 10-year (average) follow-up period [6]. Other investigators have reported a gradual decline of vision (0.032+/0.045 logMAR/year) [27], but this was not evident in the majority (72%) of our cohort [6]. ADOA has been reported to manifest during childhood, often as early as 3–5 years of age, and in a recent study 80% of individuals were symptomatic before the age of 10 years, with the average age of onset being 7 years [27]. Considering all pedigrees, the mean age at examination was 38 years (±20 years). In this cohort the average age of onset of vision impairment was 10.2+/−10.1 years [7]. Across the whole study population there was no statistical difference between the initial visual acuity measurement and visual acuity measured at a follow-up visit an average of 9.6+/−7.9 years later [6]. Furthermore there were no significant differences in the rate of visual loss between families harbouring different OPA1 mutations [6]. Thus age of onset, OPA1 mutation and time since diagnosis are not thought to be confounding factors and there was no significant difference in the mean age between groups.

### OPA1 expression, mitochondrial DNA content and mitochondrial structure in ADOA cells

**Figure 3:**
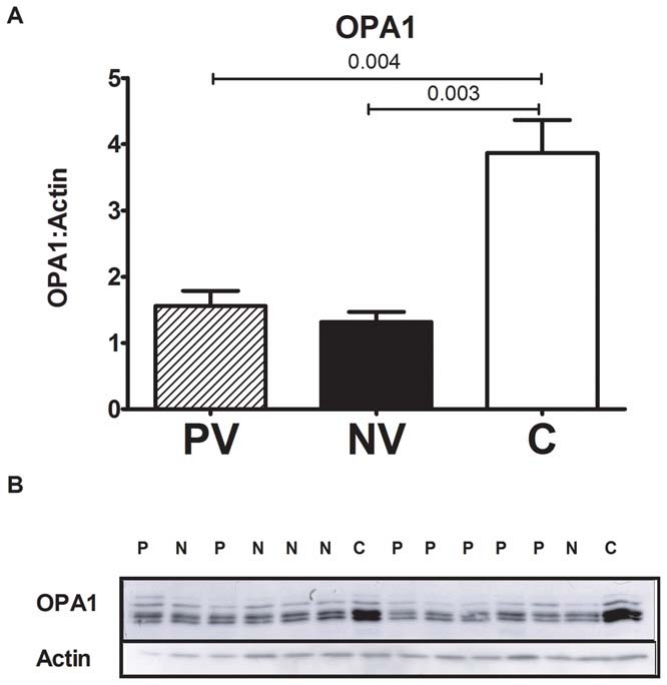
Decreased OPA1 protein levels in ADOA patient cells. A) Representative Western blots of OPA1 protein in patient and control cells. B) Quantification relative to actin shows a decrease in total OPA1 content in both normal vision and poor vision ADOA compared to controls. *** p<0.001.

OPA1 haploinsufficiency is a consequence of most ADOA-associated OPA1 mutations [7], [36]. To determine if our lymphoblast cellular model expressed OPA1 haploinsufficiency, total protein lysates from cell lines from ADOA patients and controls were examined for expression levels of OPA1 by western blotting (Figure 3B). We detected two major bands corresponding to OPA1 just above and below 90 kDa (Figure 3A), corresponding to the 92 kDa and 86 kDa isoforms previously reported by other groups [37]. Normalisation of total OPA1 content to actin showed a significant reduction in total OPA1 content by around 60% in patients with ADOA (p<0.001), with no difference in levels between patients with poor vision and normal vision (Figure 3). This confirms that OPA1 mutations result in equal levels of haploinsufficiency of OPA1 in our lymphoblast cell lines between patients with normal or poor visual acuity. We also found no difference in the processing of OPA1 isoforms between groups (data not shown). Function of the OPA1 protein appears to be modified by alternative splicing events which regulate mitochondrial morphology [12], [38], [39], [40].

OPA1 may preserve mtDNA stability, because in yeast models lacking a homologue of OPA1 [41] or in more severe cases of ADOA [25], [42] multiple deletions and depletions of mtDNA have been identified [42]. To explore possible changes in mtDNA quantity, or the presence of large mtDNA deletions in patient lymphoblasts, we visualized mtDNA by southern blotting. We did not observe any large-scale deletions in mtDNA, which would be detected as smaller fragments appearing on the southern blot (Figure S1A). There was also no significant difference in the mtDNA copy number between ADOA groups or controls (Figure S1B). The mean ratio of mtDNA/18SRNA probe signal in control patients (n = 10) was 2.91+/−0.49 (+/− SEM), 2.46+/−0.29 in ADOA patients with normal vision (n = 7) and 2.55+/−0.35 in ADOA patients with poor vision (n = 8).

Because OPA1 plays a key role in mitochondrial fission/fusion, we assessed whether OPA1 haploinsufficiency in the normal vision and poor vision ADOA cells induced structural changes to the mitochondria, or affected the mitochondrial density per cell. To investigate this we quantified mitochondrial area per cell by transmission electron microscopy (Figure S2). There was no significant difference in mitochondrial area per cell (2 way ANOVA, P>0.05) between any of the groups (Figure S2) nor were there any changes in the size distribution of individual mitochondria between groups (Figure S2). Thus in this cellular model we observe OPA1 haploinsufficiency characteristic of ADOA, without depletion of mitochondrial DNA or gross disruption of mitochondrial volume or structure.

### Impaired ATP synthesis in ADOA patients with poor vision

**Figure 4:**
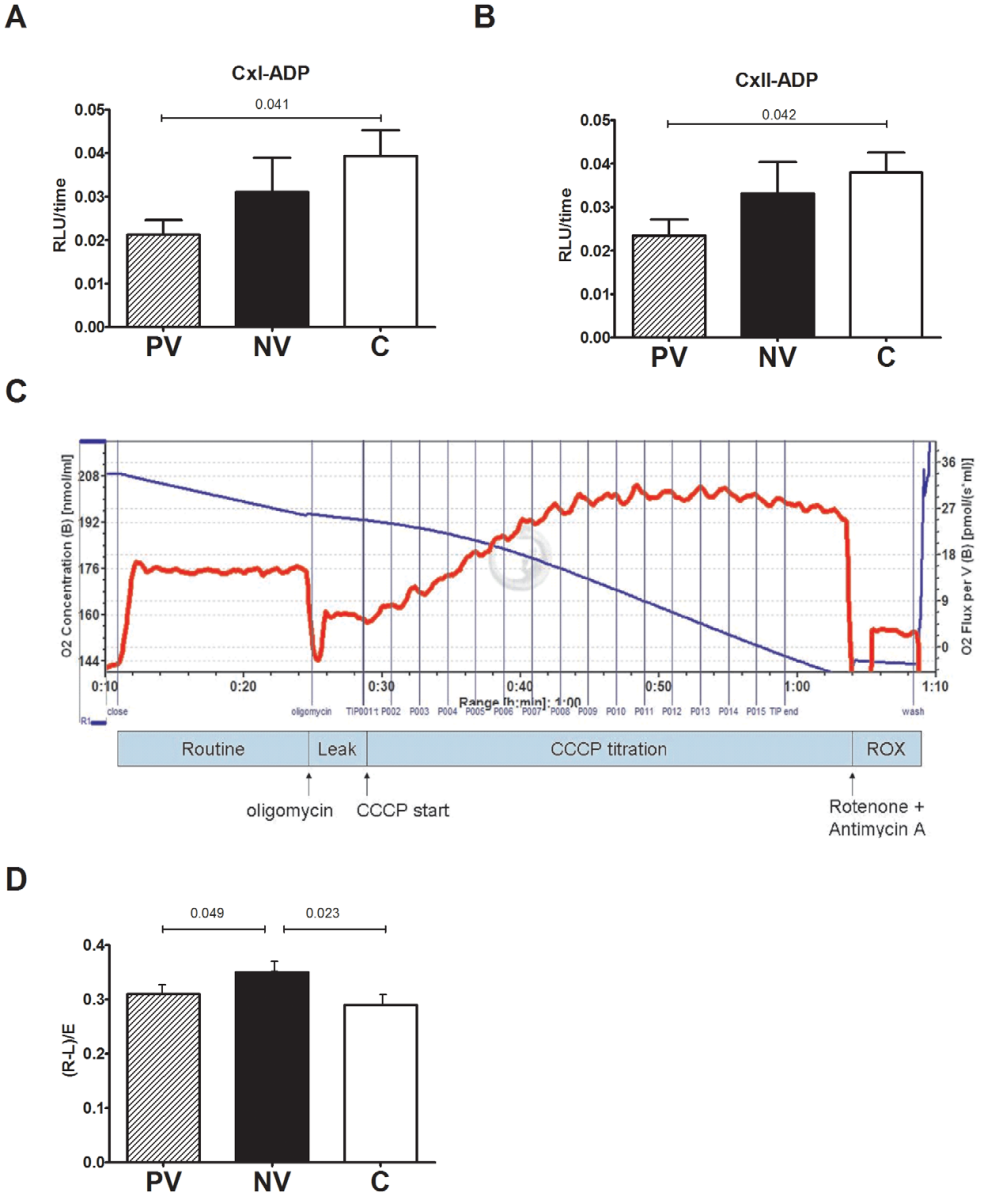
Reduced ATP production and endogenous respiration in poor vision ADOA patients. The rate of mitochondrial ATP synthesis was determined in cells treated with 50 ug/ml digitonin. ATP synthesis driven by A) complex I (glutamate plus malate) or B) complex II (succinate plus rotenone) substrates was determined. This shows a significant decrease in complex I and II driven ATP production in poor vision, but not normal vision ADOA compared to controls. C) An example trace showing respiration of intact cells on endogenous substrates (routine), leak (oligomycin inhibited), maximum electron transport capacity (CCCP titration) and residual oxygen consumption (ROX). D) Cell respiration *in vivo* at intracellular, non-saturating ADP levels showed normal vision ADOA cells have a higher routine respiration rate.

We hypothesised that ADOA patients with poor vision would demonstrate an impairment in mitochondrial energy production. To investigate mitochondrial energy production in ADOA cells we measured the rate of mitochondrial ATP synthesis in digitonin permeabilised cells provided with complex I substrates (glutamate + malate) or complex II substrate (succinate + rotenone) in the presence of ADP. We found a significant decrease in both the complex I (Figure 4A) and complex II driven ATP synthesis rates (Figure 4B) in poor vision ADOA patients compared to controls. The rates of complex I driven ATP synthesis in poor vision ADOA patients were decreased to 49% of controls (p<0.05), and the complex II driven ATP synthesis rate was 57% of controls (p<0.05). Normal vision patient cells in contrast did not show significantly lower ATP production rates.

We also investigated whether this defect in ATP synthesis correlated with impaired mitochondrial oxygen consumption, which provides an indirect measurement of overall electron transport chain activity via endogenous mitochondrial respiration. Measurement of the endogenous respiration state in intact cells revealed a small but significant increase in the respiration rate of normal vision ADOA cells compared to poor vision ADOA cells and controls (Figure 4C). Together with the relative preservation of ATP production rate in normal vision patients, this suggests that this normal vision patient group is able to better upregulate respiration to partially compensate an ATP production deficit due to OPA1 haploinsufficiency.

### Increased oxidative phosphorylation enzyme activity associated with normal vision ADOA

A detailed OXPHOS enzymology study was undertaken to investigate whether modulation of this pathway may correlate with the observed increase in normal vision patient cell respiration and partial ATP production rescue. The specific activities of OXPHOS enzyme complexes I, II, II+III, III, IV and the Kreb's cycle enzyme citrate synthase (CS) were measured in mitochondrial fractions from patient cells.

**Figure 5:**
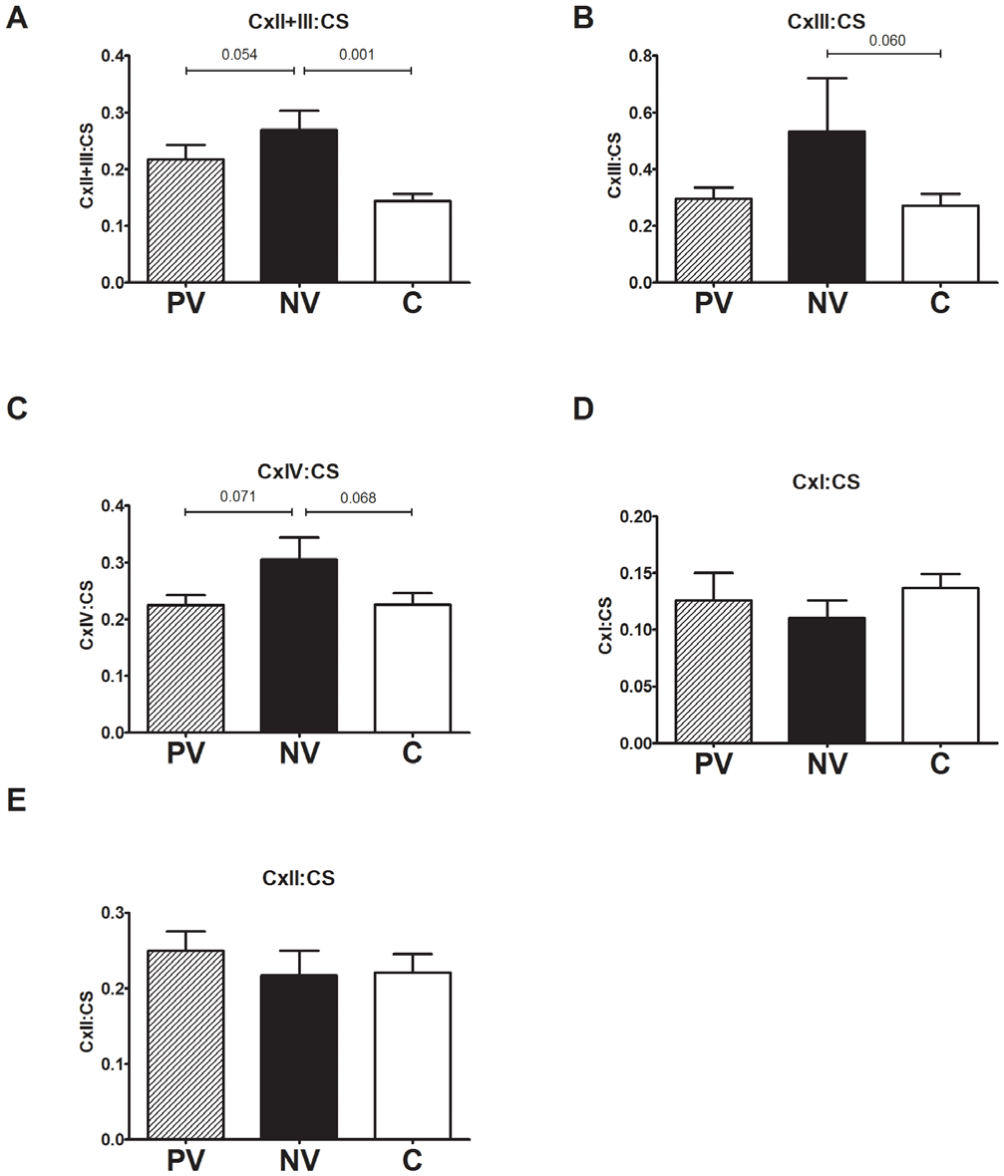
Oxidative phosphorylation complex activities are increased in normal vision patients. Lymphoblast mitochondria were used to determine the specific activity of OXPHOS enzymes. A) cytochrome c reduction at 550 for complex II+III linked assay, B) cytochrome c reduction at 550 for complex III, C) oxidation of ferrocytochrome c at 550 nm for complex IV, D) NADH oxidation at 340 nm for complex I and E) DCIP reduction at 600 nm for complex II. The data was normalised to the citrate synthase activity of each mitochondrial sample. Data are average +/− SEM. (P) poor vision (n = 8), (N) normal vision (n = 7), (C) controls (n = 21) for 3 independent experiments. There was a significant increase in the complex II+III activity in normal vision ADOA, with trends of increased complex III and IV when compared to poor vision ADOA.

The specific activity of OXPHOS complexes II+III, (a linked assay that relies on endogenous ubiquinone) was significantly increased in normal vision patient mitochondria compared with controls (Figure 5A). Similar trends (not reaching statistical significance) were seen for complex III and IV (Figure 5B, C). This trend was not seen for complexes I or II (Figure 5D, E). This indicates that the higher endogenous respiration in normal vision ADOA may be accounted for by an increase in the specific activity of certain OXPHOS enzymes.

### Increased mitochondrial protein expression in normal vision ADOA cells

**Figure 6:**
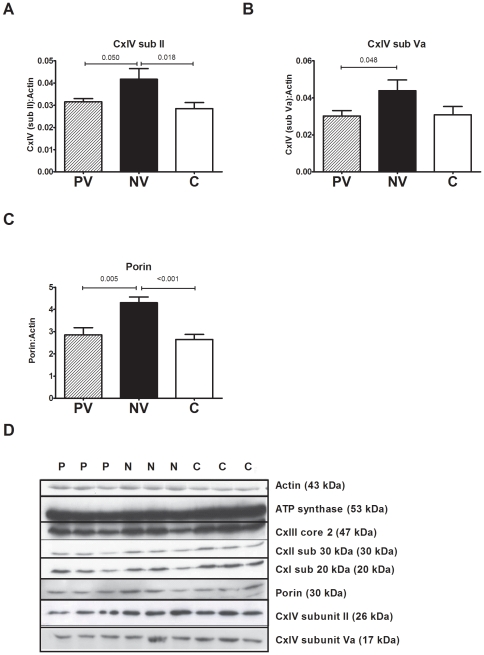
Western blot analysis of complex IV and porin expression in ADOA cell lysates show increases in normal vision ADOA patients. Total cellular protein was loaded onto a 12% acrylamide gel, transferred and probed with a suite of antibodies (Table S3). A,B) There was a significant increase in CxIV subunit II and IV content as well as C) porin in normal vision ADOA compared to poor vision ADOA and controls. However there was no change in expression of other OXPHOS enzymes including Complex I, Complex II, Complex III and ATP synthase (data not shown). D) A representative blot. Data is average +/− SEM. (P) poor vision (n = 8), (N) normal vision (n = 7), (C) controls (n = 21) for 3 independent experiments.

To further explore the changes observed in OXPHOS complex activities levels of OXPHOS proteins were visualized by western blotting (Figure 6A). The levels of two complex IV subunits, both nuclear and mitochondrial DNA encoded, were significantly increased in normal vision ADOA compared to poor vision ADOA and controls (Figure 6A and B, P<0.05). In normal vision ADOA the expression levels of the nuclear encoded CxIV subunit (Va) was increased to 145% and the mitochondrial encoded CxIV subunit (II) to 139% of poor vision ADOA patient cells. We also recorded a 145% increase in the expression of porin in patients with normal vision (p<0.01), compared to poor vision and controls (Figure 6C). No differences were seen among the groups for subunits of complex I, II or III (data not shown).

### Gene expression levels by RT-PCR and western blotting

We explored the mechanism of the increase in OXPHOS function by measuring expression levels of key proteins in the mitochondrial biogenesis pathway. In particular we measured both RNA and protein levels of PGC-1α, TFAM and NRF1. PGC-1α commercial antibodies are widely criticised for lacking specificity. We found the same problem and were not confident in drawing conclusions from the antibody tried. Measuring PGC1 RNA levels by real-time PCR also proved difficult, we were not able to quantify expression of PGC1 in any of our lymphoblast samples due to low signal. This finding is supported by another study which found levels of PGC1 expression in lymphoblast cells to be very low [43].

**Figure 7:**
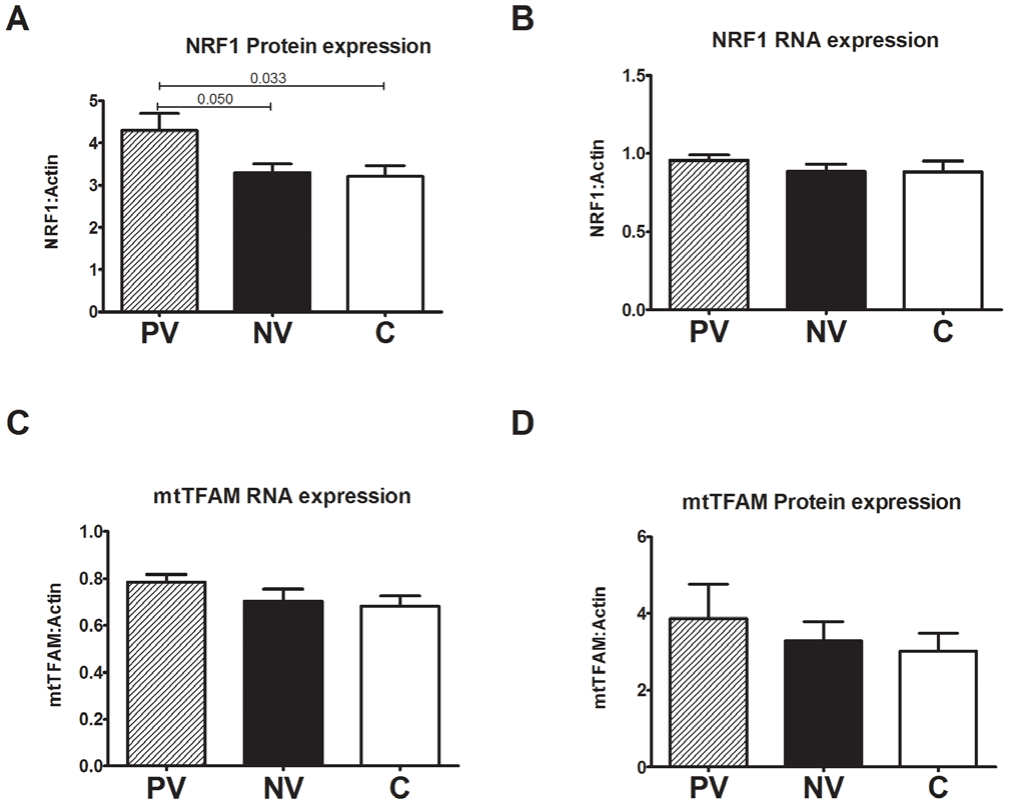
Gene and protein expression levels of mitochondrial biogenesis markers. Total cellular protein was loaded onto a 12% acrylamide gel, transferred and probed with antibodies against NRF1, mtTFAM and b-actin (Table S3). Gene expression was measured by RT-PCR using probes for NRF1, mtTFAM and b-actin A) There was a significant increase in NRF1 protein expression content in poor vision ADOA compared to normal vision ADOA and controls. However there was no change in gene expression of C) NRF1 or gene expression of B) mtTFAM or D) protein expression of mtTFAM. Data is average +/− SEM. (P) poor vision (n = 8), (N) normal vision (n = 7), (C) controls (n = 21) for 3 independent experiments.

However when we measured expression levels of mitochondrial biogenesis proteins that are activated downstream of PGC1 to drive mitochondrial DNA transcription (TFAM) and nuclear mitochondrial protein transcription (NRF1) we found a significant increase in the protein level (by western blotting) of NRF1 in the PV group (Figure 7), however mtTFAM was not significantly upregulated. The RNA expression levels were not significantly changed for either of these genes but did follow the same trend.

## Discussion

In this study we identified variations in OXPHOS capacity between ADOA patients who maintain or lose vision. We show that lymphoblasts derived from ADOA patients with normal vision maintain ATP synthesis rates that are above those of poor vision patients. The relative preservation of mitochondrial ATP production in normal vision ADOA patient lymphoblasts compared to poor vision patients may occur via compensation of OXPHOS function. We found higher cell respiration rates together with increased distal OXPHOS complex activity and protein expression. This signature of increased mitochondrial function in normal vision patients has not been observed before and may point toward potential compensatory pathways associated with preserved vision.

The decrease in OXPHOS capacity in poor vision patients supports other published reports. These other studies were in patients with clinical signs of optic atrophy and vision impairment [18], [26], [43], including one study in which 75% of patients had visual acuity worse than 6/18 [26]. In these studies, patients presenting with clinical symptoms showed a reduced ATP content and ATP synthesis across different OPA1 mutations including the c.2709-2711delTTAG in exon 27 [26], the R445H mutation [43] and in five other OPA1 mutations [18]. In patients with multi-system disorders associated with OPA1 mutations (ADOA+), the degree of energetic impairment was significantly more pronounced than in patients with ‘typical’ OPA1 mutations [44], [45]. This precedent exists with the previously recognized LHON+ syndrome in which OXHOS studies demonstrated more severe biochemical defects than associated with the ‘typical’ LHON complex I mtDNA gene mutations [46]. When considered in context with other reports [25], [44] our data indicate that OXPHOS capacity may be a key pathway altered in ADOA patients with vision loss, in whom increasing dysfunction leads to involvement of other vulnerable neuronal populations resulting in more complex phenotypes.

We initially hypothesised that alterations in oxidative phosphorylation function may contribute to the decreased ATP synthesis observed in our poor vision patients. In these patients we did not detect significant differences in the OXPHOS enzyme complex activities when compared to controls. This supports a previous study that examined OXPHOS activities in six clinically affected (i.e. poor vision) ADOA patient lymphoblast cell lines. Mayorov *et al* (2008) found no difference in complexes III, IV and citrate synthase between their ADOA patients and controls with only a slight decrease in the complex I activity. Further polarographic analysis of respiration in isolated mitochondria also showed no decrease compared to controls [47], which was also observed in another study by Spinazzi *et al* [48], with both these studies agreeing with our finding of normal intact cell respiration in our poor vision patient cells. These studies did not include ATP production assays. Thus our findings of no changes in OXPHOS rates between our PV and C patients support Mayorov's data. However our approach of sampling from the extremes of visual acuity adds the novel finding of *increased* cell respiration, OXPHOS capacity and relatively preserved mitochondrial ATP production in patients who maintain normal visual acuity. An increased mitochondrial respiratory capacity (increased OXPHOS and respiration) may be a response of the cell to maintain ATP synthesis, and the failure to respond to this (in the PV group) may result in a decline in ATP synthesis.

We further explored the basis of these oxidative phosphorylation differences by western blotting of mitochondrial proteins with antibodies directed against complex IV nuclear- and mtDNA-encoded subunits as well as key subunits of oxidative phosphorylation proteins. In support of the oxidative phosphorylation enzymology data, both nuclear- and mtDNA-encoded complex IV subunits were significantly increased in the normal vision patients. This may point to key changes in mitochondrial biogenesis or gene regulation that allow for upregulation of certain OXPHOS complexes, and this upregulation may mediate protection against decline in visual acuity in our NV patients. We found that NRF1 protein levels were modestly increased in the PV group, with a non-significant trend for higher TFAM protein levels, and similar non-significant trends for higher NRF1 and TFAM transcript levels measured by real-time PCR. These results suggest that the NV patients are able to increase some aspects of OXPHOS function independently of the PGC1 axis. We speculate that the PV patients' cells may be less able to compensate in the same way, but are attempting to upregulate the PGC1 pathway.

A limitation of this study is the sample size of our patient groups. We limited the number of families involved in this study to minimise the number of OPA1 mutations included. The majority of patients chosen were from the largest ADOA pedigrees with a wider range of acuities among siblings. Furthermore not all of these extensively studied pedigrees shared the same range of VA, one pedigree with 100% penetrance had an overall average poorer visual acuity than other pedigrees, as discussed previously [7]. We sampled patients at the extremities of VA to allow the most significant biochemical differences to be detected. Within this study there were two younger patients included in the NV group. ADOA has been reported to manifest during childhood, often as early as 3–5 years of age, and in a recent study 80% of individuals were symptomatic before the age of 10 years, with the average age of onset being 7 years [27].

The relationship between OPA1 haploinsufficiency and mitochondrial structure and function, and neuronal viability has been extensively demonstrated in *Caenrhabditis elegans*, drosophila and rodent models. Mutations of OPA1 orthologues resulted in impaired oxidative phosphorylation [20], [49] together with a marked increase in susceptibility to oxidative stress [20], increased reactive oxygen species production and mitochondrial fragmentation [23]. These OPA1 mutations contributed to decreases in the number of RGCs [19] impaired visual function [21], [22] and lower visual evoked potential measurements [50]. These were not associated with any mtDNA mutations or COX deficiencies [24]. These models mimic the human disease, with progressive visual failure due to loss of RGCs and suggest that OPA1 haploinsufficiency may lead to increased oxidative stress, mitochondrial dysfunction and eventual loss of RGCs.

Our studies suggest that genetic modifiers influence the course of RGC loss in OPA1-linked ADOA. The mitochondrial haplogroup has been excluded as a disease modifier in ADOA [32], [33]. Our pedigrees also argue against mtDNA haplogroup having a strong influence on phenotype, since we show strong discordance within sibships that share haplotypes.

Identifying the genetic variants that contribute to the OXPHOS compensation we observed in patients with normal vision may permit new methods for predicting individuals most at risk of losing vision, and also identify novel therapeutic targets for preserving RGCs in primary optic neuropathies including glaucoma, in which mitochondrial dysfunction may also have a role in pathogenesis [51], [52].

## Materials and Methods

### Patient grouping and selection

For this study we recruited ADOA patients from the six largest of 17 pedigrees within our population cohort of 158 subjects [7]. These individuals had one or more of six different OPA1 mutations including: exon 27 2708delTTAG, exon 9&11 937AG>TA and 1126A>G, exon 19 1298G>A, intron 9 985-1g>a and exon 2 112C>A and deletion of a copy of the entire OPA1 gene (Figure 1B). Across the six pedigrees there were a total of 119 patients carrying OPA1 mutations. These pedigrees have been extensively described elsewhere [7].

To determine whether there is an association between visual acuity and mitochondrial function, we ranked patients according to their best-corrected visual acuity (BCVA) and then derived lymphoblast lines from the ADOA patients with the upper and lower percentiles of visual acuity. (Figure 1A, Table S1, and Table 1). This generated two groups of ADOA patients that were used for comparison - the “normal” vision group (NV)” with normal optic disc appearance and visual acuity greater than 6/9 in the best eye (n = 7) and the “poor vision” group (PV)” with visual acuity less than 6/36 (n = 8) in the presence of clear optic disc pallor. Patients with visual acuity in the mid-range (less than 6/9 but better than 6/36) were not included for mitochondrial studies.

**Table 1:** ADOA patient summary.

Group	OPA1 mutations*	VA range	Age	Gender
Poor Vision - PV	1×A, 2×B, 3×C, 1×D, 1×F	6/36-count fingers	25–60	3M 5F
Normal Vision - NV	3×C, 1×D, 3×E, 1×F	6/5–6/9	10–66	4M 3F
Control - C	non mutation controls	Normal vision, unknown	6–63	13M 8F

*A = 2Arg38 STOP (c112C>T), B = exon10 985-1G>C, C = exon27 4bpdel 2708delTTAG, D = 1798G-T Glu600STOP exon1, E = entire OPA1 del, F = exon9 937A-G & 938T-A & exon11 1126A-G.

For further comparison, we employed lymphoblast lines from nineteen subjects from the CEPH (Centre d'Etude du Polymorphisme Humain, Utah residents with ancestry from northern and western Europe) HAPMAP population at the Corriell cell repository [53], which has been used as a reference European American population [54] in both functional and genomic studies [55], [56], [57]. We also included one non-mutation carrying ADOA family member who was a genetically unrelated marriage partner.

### Clinical assessment and ethics statement

ADOA subjects were recruited from different clinical centres across Australia. The study conformed to the tenets of the Helsinki Declaration, and informed written consent was obtained from all participants prior to being included in the study. The protocol for the research project was approved by the local Ethics Committee (Royal Victorian Eye and Ear Hospital, Melbourne, Australia) and the work was undertaken conforming to the provisions of the Declaration of Helsinki in 1995 (as revised in Tokyo 2004). All subjects gave written informed consent. A 10 ml blood sample was collected from each patient. Participants were examined using Snellen acuity charts or logMAR charts for BCVA. When the measurement was recorded in Snellen acuity, the reading was then converted to the logMAR equivalent for analysis [58]. The Farnsworth–Munsell 100 Hue test was used to test colour vision and visual fields were assessed with Humphrey 24-2 SITA automated perimetry (Humphrey Field Analyser II, Zeiss-Humphrey, Dublin, CA, USA). Stereoscopic optic disc photography was performed with a Nidek Stereo Fundus 3-Dx/F camera (Nidek, Gamagori, Japan). Analysis of the optic cup area, vertical cup-to-disc ratio and neuro-retinal rim area was performed stereoscopically using custom software (StereoDx) with a Zscreen (StereoGraphics, Beverly Hills, CA, USA) [59], [60].

### EBV transformation of B-lymphocytes and lymphoblast culture

B-cell lines were generated using established techniques [35]. Briefly, blood stored in EDTA blood tubes (Greiner Bio-one) for up to 3 days at room temperature was successfully used to isolate lymphocytes and transform with Epstein-Barre Virus. Transformed lymphoblasts were maintained in RPMI (2 mg/ml glucose, Gibco) with 15% heat-inactivated fetal calf serum (56°C for 30 minutes, Gibco) and penicillin/streptomycin (Sigma). Culture flasks were kept on an angle in a humidified 37°C/5% CO_2_ incubator. Lymphoblast lines were archived at passages 3 to 4 and used for experiments up to passage 25.

### Transmission Electron Microscopy (TEM)

Lymphoblasts were fixed using 2.5% glutaraldehyde, stained and embedded for TEM using standard protocols. Sections were viewed under a Phillips CM10 electron microscope and images captured on film. Images were scanned and adjusted to a pre-calibrated scale bar and mitochondrial, nuclear and cytoplasmic area and mitochondrial lengths and widths measured using ImageJ software [61].

### Mitochondrial isolation and OXPHOS enzymology

Intact mitochondria were isolated from lymphoblasts following gentle lysis with digitonin as previously described (Trounce et al 1996). We are able to obtain high levels of mitochondrial purity using this isolation technique, determined by western blotting using mitochondrial, endoplasmic reticulum and lysosomal specific markers (data not shown). Aliquots of mitochondrial fractions were stored at −80°C so that OXPHOS assays could be performed on complete groups to minimize inter-assay variation. Prior to OXPHOS assays, mitochondria were disrupted by sonication. Isolates were diluted to 1 mg/ml in isolation buffer on ice, and disrupted on an ice slurry using 4 pulses over 3 seconds at power 1.5 (∼4 watts)(Misonix Microson Ultrasonic homogeniser - Misonix, USA, fitted with a 3.2 mm microprobe). OXPHOS complexes I, II, III, II+III and IV, and citrate synthase assays were performed as previously described [62] except that a single beam spectrophotometer was used (Cary 3000 spectrophotometer, Varian, USA).

### Mitochondrial DNA analysis

Probes to span the entire human mtDNA genome were produced using 5 PCR fragments as previously described [63], [64] and are listed in Table S2. A probe representing a fragment of the nuclear 18sRNA gene was produced by PCR as described previously [65].

DNA was extracted from cells using the Qiagen DNAse extraction kit according to the manufacturer's protocol. 5 µg of DNA was cut using BamHI (NEB, according to the manufacturer's protocols), which has a single restriction site in most human mtDNAs. The restricted DNA samples, along with molecular weight marker (500 ng lambda phage DNA digested with *HindIII*), were electrophoresed on a 0.6% (w/v) agarose (Scientifix) gel +10 µg/ml ethidium bromide in TBE buffer overnight at 40 V. Following transfer to Hybond N+ nylon membrane (Amersham) and probing with 32P-labelled hybridization probes, hybridizing bands were visualized on a phosphoimager screen. Images were quantified using ImageJ software [61].None of the ADOA patients in this study linked in with any of the 60 Leber's Hereditary Optic Neuropathy (LHON) matrilineal pedigrees in Australia.

### Western Blot

Total cell or mitochondrial protein lysates (20–50 µg) were electrophoresed on SDS-PAGE reducing gels (8–14% acrylamide depending on separation required, Biorad). Proteins were transferred to Hybond N+ nylon membranes (Amersham, GE Healthcare), membranes blocked with 5% (w/v) skim milk in PBS for 1 hour at room temperature, and washed in 0.05% tween-20/PBS three times. Membranes were then probed with primary antibodies in 5% BSA/PBS overnight (Table S3) at 4°C, washed in 0.05% tween-20/PBS three times and probed with appropriate horse radish peroxidase conjugated secondary antibodies. Primary and secondary antibodies used in this study are listed in Table S3. Following development with ECL detection reagents (Amersham, GE Healthcare), membranes were exposed to autoradiography film (Amersham, GE Healthcare). Autoradiography films were scanned to .tiff files using Arcsoft Photos Studio 5 (Arcsoft Inc, 2003) and bands were quantified using Image J software [61].

### Mitochondrial ATP synthesis in cultured lymphoblasts

The amount of cellular ATP was determined by using a luciferin/luciferase assay as previously described [66], [67], [68] with some modifications. The measurements of mitochondrial ATP synthesis were performed in cells grown in RPMI with 2 mg/ml glucose and 15% heat inactivated fetal calf serum. Cells were resuspended (7×10^6^ cells/ml) in buffer A (10 mmol/L KCl, 25 mmol/L Tris-HCl, 2 mmol/L EDTA, 0.1% bovine serum albumin, 10 mmol/L potassium phosphate, 0.1 mmol/L MgCl_2_ (pH 7.4)), kept for 15 minutes at room temperature, and then incubated with 50 µg/mL digitonin for 1 minute. After centrifugation, the cell pellet was resuspended in the same volume of buffer A and aliquots used to measure ATP synthesis. Measurements were performed at 30°C in a 96-well luminescent platereader (Fluorostar Optima, BMG instruments) in white-walled, white-based 96-well plates. 50 µl aliquots of digitonin permeabilised cells were incubated for 5 minutes with 50 µl of 25× diluted FLAAM luciferase/luciferin reagent (Sigma) either with 5 mmol/L malate plus 5 mmol/L glutamate (measuring ATP production via complex I) or with 10 mmol/L succinate plus 2 µg/mL rotenone (measuring ATP production via complex II). Parallel samples were also included with 10 µg/ml oligomycin to inhibit ATP synthase. Luminescence was monitored for 5 minutes prior to the addition of 0.2 mM ADP, then luminescence was monitored for 30 minutes. The rate of ATP synthesis was linear for over 30 minutes, was dependant on cell density and substrate concentration, and the luminescence signal was eliminated by either the addition of oligomycin (ATPase inhibitor) or by not providing substrates (data not shown).

### Endogenous respiration in intact lymphoblasts

Cell respiration *in vivo* is regulated according to intracellular, non-saturating ADP levels. We used the Oxygraph-2 k high resolution respirometer (Oroboros Instruments, Innsbruck Austria) because of the superior resolution of slow oxygen consumption rate transitions the machine can measure compared with conventional oxygen electrodes [69]. Following saturation of RPMI growth media (RPMI with 2 mg/ml glucose and 15% heat inactivated fetal calf serum) at ambient oxygen for 30 min at 30°C, cells were added to the chamber at 7×10^5^ cells/ml and respiration measured (R). Non-phosphorylating leak state of respiration (L), mainly caused by compensation of the proton leak after inhibition of ATP synthase, was then induced by the addition of oligomycin (2 µg/ml). Uncoupled maximal respiration (UC) was then measured by addition of CCCP (Carbonyl cyanide 3-chlorophenylhydrazone, 5 µM). The respiratory control ratio (R-L/UC), which reflects the state of activation of cellular respiration according to routine endogenous ATP demand, was derived from these values [70], [71]. Oxygen consumption was calculated using DataGraph software (Oroboros Instruments).

### Gene expression by real-time PCR

For each cell line 3 independent cultures of each cell line grown under identical culturing conditions in RPMI (2 mg/ml glucose, 15% HI-FCS, PSF). Isolated cells were used for RNA extraction using Qiagen RNA isolation kit (Qiagen, Hilden, Germany). cDNA was generated from equal amounts of total RNA per sample using Applied Biosystems High Capacity RNA-to-cDNA kit (Applied Biosystems). Equal volumes of cDNA were transferred into the real-time PCR reaction. All Taqman Gene Expression Assay (Applied Biosystems) probes used spanned an exon junction to prevent amplification of genomic DNA. Commercially available probes included NRF1 (Hs00192316_m1: FAM dye), mtTFAM (Hs01082775_m1: FAM dye), PGC1α (Hs01016719_m1: FAM dye) and b-actin as a housekeeper reference gene (Hs99999903_m1: VIC dye, primer limited), all which were provided as 20× solutions. Standard curves were generated for all probes by a 1∶10 serial dilution of a control cDNA sample. Reaction efficiencies were >95% for all probes except PGC1, with R-squared values exceeding 0.97. Real-time PCR reactions were run in duplex reactions with the gene of interest probe and the housekeeping probe present in each reaction vial. Each 15 µl reaction consisted of: 7.5 µl of 2× Taqman Mastermix (Applied Biosystems), 0.75 µl of 20× gene of FAM labelled “gene of interest” probe (NRF1, PGC1α or mtTFAM), 0.75 µl of 20× gene of VIC labelled, primer limited “housekeeper” probe (b-actin), 1 µl of diluted cDNA and water to 15 µl. Negative controls included water only and reverse transcription reactions without RNA present. The real-time reactions were run using a RotorGene Q instrument (Qiagen, Hilden, Germany) and the following cycles: 1 cycle of 50°C for 2 minutes then 95°C for 10 minutes followed by 40 cycles of 95°C for 15 seconds then 60°C for 60 seconds. Data acquisition for both probes occurred during the 60°C cycle. The PCR products were verified by gel electrophoresis. To avoid inter-assay variability all samples analysed were performed with the same cDNA dilution, with an internal control sample present in each assay. To further verify the accuracy of real-time PCR reactions the “gene of interest” and b-actin probes were amplified in the same tubes, as well as each probe individually. The intra-assay and intra-well variation was low for all probes. CT values for each sample were analysed by the Rotor-gene 6000 software (Version 1.7, Corbitt Research) and the delta-delta analysis algorithm and the threshold values determined from each probe's standard curves. Expression levels were measured in triplicate and expressed as relative quantification of the gene of interest to b-actin.

### Statistical analysis

All data are presented as mean ± SEM. Statistical analyses were performed using a commercially available software package. (SPSS ® v 15.00, SPSS Inc., Chicago, IL). Two-tailed Student's t-test with assumed equal variance and two way ANOVA with Tuckey's post-test was used for comparison between groups with alpha of 0.05.

## Supporting Information

Figure S1 **Mitochondrial DNA content in ADOA patient cells.** A) Southern blot of BamH1 digest of total cell DNA probed with 5 overlapping mtDNA probes, while the lower panel shows the 18S rDNA probe labelling for nuclear gene quantification. B) There was no significant change in mtDNA levels between normal vision ADOA (NV), poor vision ADOA (PV) or controls (C), nor were there large-scale deletions indicated by lower molecular weight mtDNA bands.
(TIF)

Figure S2 **Transmission Electron Microscopy images of ADOA and control cells.** A) EM micrographs of PV ADOA, NV ADOA and controls. Bar scale: 1 µm. B) Mitochondrial area per cell was quantified and divided by the cytoplasmic area. There was no significant difference in mitochondrial area per area of cytoplasm between any of the groups. Per group >10 cells were scored for total mitochondrial, nuclear and cytoplasmic volume. C) Mitochondrial area was plotted against frequency (% total mitochondria) to display the size distribution of mitochondria per cell. Per group >150 mitochondria were measured.
(TIF)

Table S1 **Table of patient characteristics.** Patients were categorised into the following groups: 1) “normal vision ADOA” where visual acuity was greater than 6/9, 2) “poor vision ADOA” where visual acuity was less than 6/36, or 3) non mutation carrying family member controls or from the well described pedigrees from the HAPMAP population at the Corriell cell repositories (The International HapMap Project: Nature). Patient characteristics including OPA1 mutation and visual acuity for both the left and right eye are shown (CF = count fingers).
(DOC)

Table S2 **Primer pairs used to produce PCR probes spanning entire human mtDNA genome.** Primers were designed for the six generated PCR fragments to cover the entire mtDNA genome. Numbers in brackets indicates position alignment to human mtDNA genome.
(DOC)

Table S3 **Antibodies used for western blotting.** A list of commercially available antibodies that were used for western blotting.
(DOC)
