# Supplementary figures and images for: Mitochondrial Oxidative Phosphorylation Compensation May Preserve Vision in Patients with OPA1-Linked Autosomal Dominant Optic Atrophy
Source: PLoS One. 2011 Jun 22;6(6):e21347. doi: 10.1371/journal.pone.0021347 (PMC3120866; doi:10.1371/journal.pone.0021347)

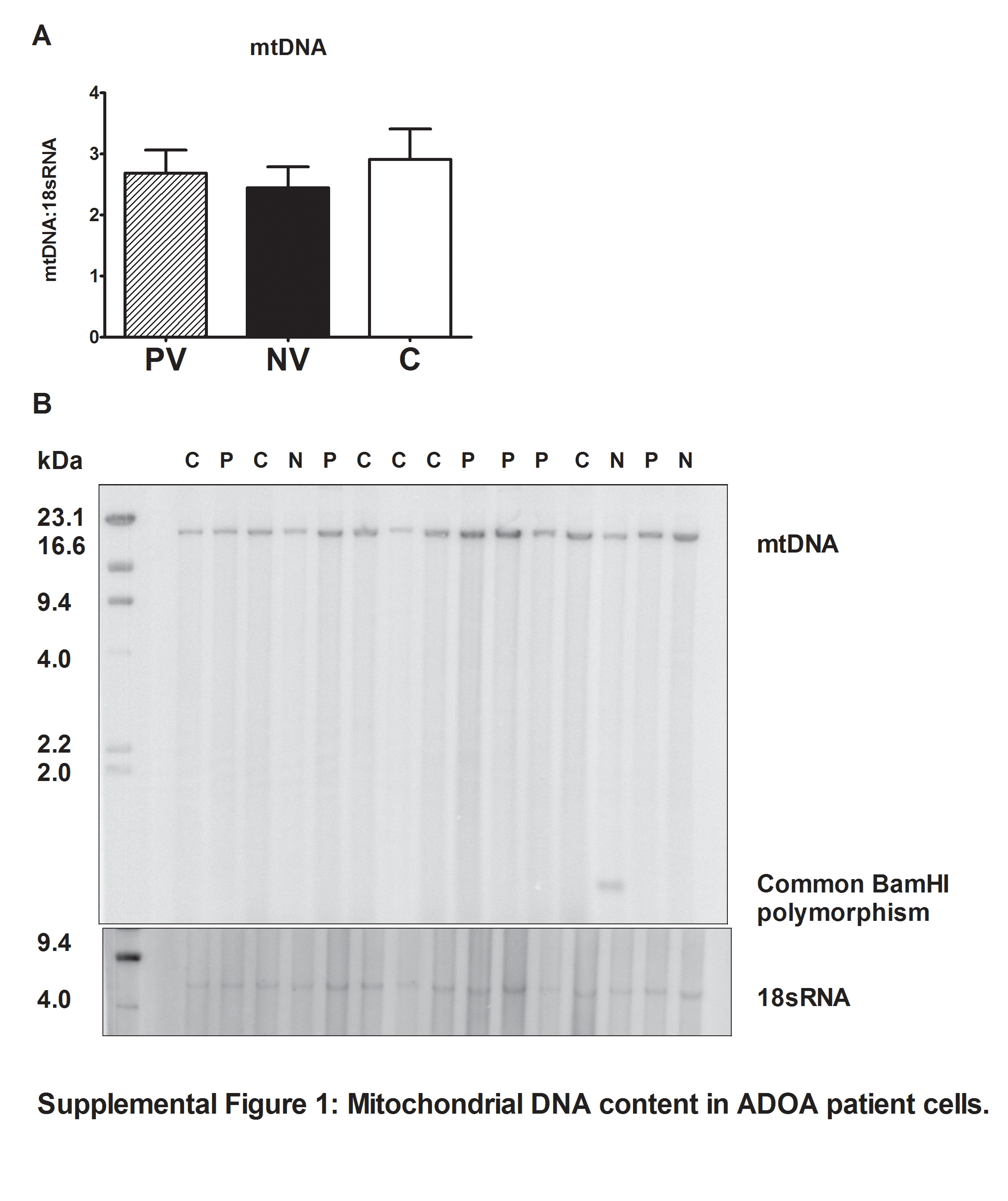

Supplement: Figure S1 — Mitochondrial DNA content in ADOA patient cells. A) Southern blot of BamH1 digest of total cell DNA probed with 5 overlapping mtDNA probes, while the lower panel shows the 18S rDNA probe labelling for nuclear gene quantification. B) There was no significant change in mtDNA levels between normal vision ADOA (NV), poor vision ADOA (PV) or controls (C), nor were there large-scale deletions indicated by lower molecular weight mtDNA bands. (TIF) [file pone.0021347.s001.tif]

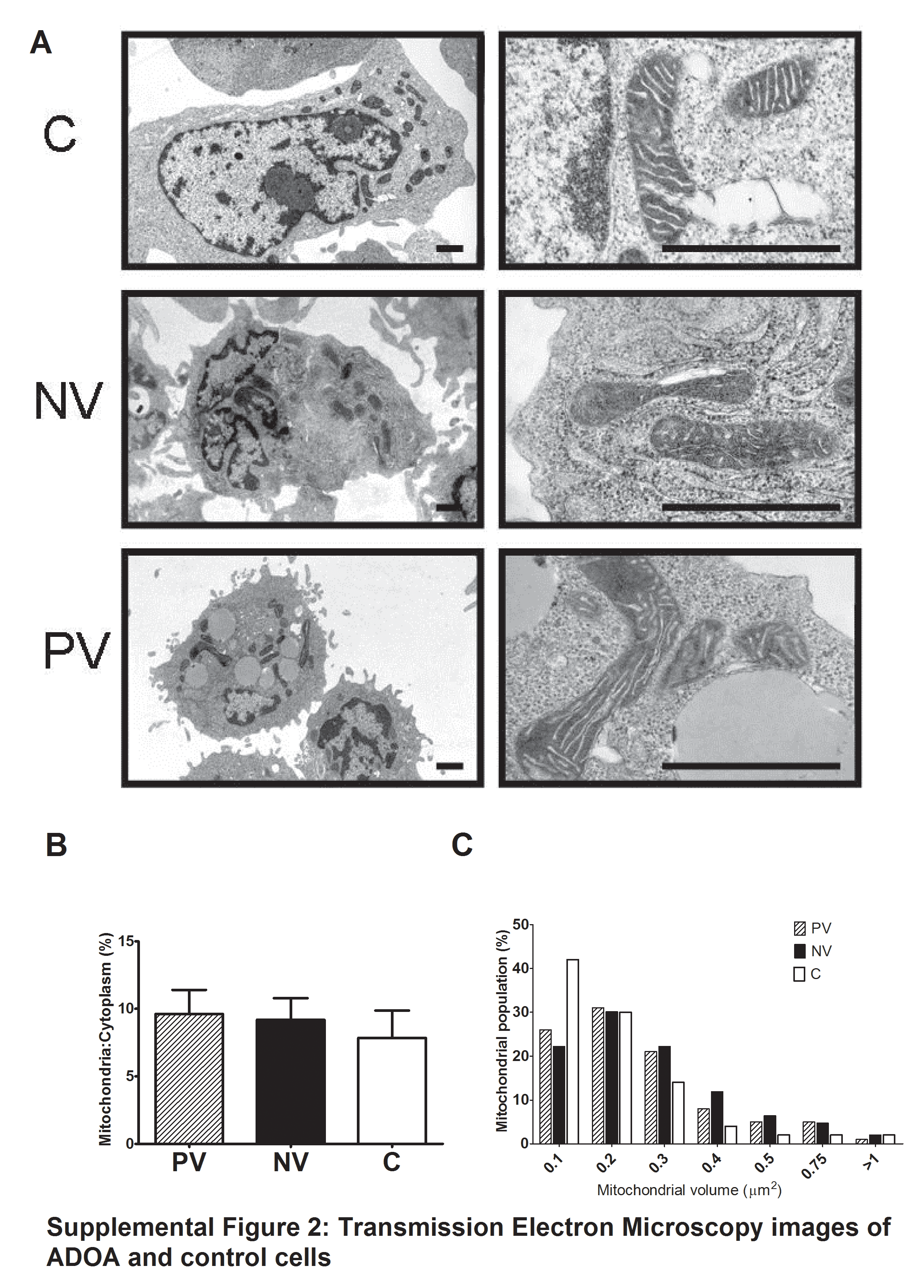

Supplement: Figure S2 — Transmission Electron Microscopy images of ADOA and control cells. A) EM micrographs of PV ADOA, NV ADOA and controls. Bar scale: 1 µm. B) Mitochondrial area per cell was quantified and divided by the cytoplasmic area. There was no significant difference in mitochondrial area per area of cytoplasm between any of the groups. Per group >10 cells were scored for total mitochondrial, nuclear and cytoplasmic volume. C) Mitochondrial area was plotted against frequency (% total mitochondria) to display the size distribution of mitochondria per cell. Per group >150 mitochondria were measured. (TIF) [file pone.0021347.s002.tif]
