# Supplementary material for: Mitochondrial Oxidative Phosphorylation Compensation May Preserve Vision in Patients with OPA1-Linked Autosomal Dominant Optic Atrophy
Source: PLoS One. 2011 Jun 22;6(6):e21347. doi: 10.1371/journal.pone.0021347 (PMC3120866; doi:10.1371/journal.pone.0021347)
Supplement: Table S1 — Table of patient characteristics. Patients were categorised into the following groups: 1) “normal vision ADOA” where visual acuity was greater than 6/9, 2) “poor vision ADOA” where visual acuity was less than 6/36, or 3) non mutation carrying family member controls or from the well described pedigrees from the HAPMAP population at the Corriell cell repositories (The International HapMap Project: Nature). Patient characteristics including OPA1 mutation and visual acuity for both the left and right eye are shown (CF = count fingers). (DOC) [file pone.0021347.s003.doc]

| **OPA1 mutation** | **Gender** | **Age** | **VA Right** | **VA Left** | **Fields** | **Discs** | **Colour V** |
| --- | --- | --- | --- | --- | --- | --- | --- |
| **Poor Vision - PV** | | | | | | |  |
| Exon 2 112 C>A | m | 25 | 6/60 | 6/36 | N/A | Pale, mild pseudocupping | N/A |
| Intron 9 9 985-1 G>A | m | 38 | 6/60 | 6/48 | N/A | N/A | N/A |
| Exon 27 2708delTTAG | f | 41 | 6/30 | 6/24 | Mild central scotoma | Pale, mild pseudocupping | RE TES 338 TPES 181; LE TES 457 TPES 214 |
| Intron 9 9 985-1 G>A | f | 46 | 3/60 | 3/60 | N/A | N/A | N/A |
| Exon 27 2708delTTAG | f | 52 | 3/24 | 2/30 | Bilateral centrocecal scotoma | No pallor | Severe errors |
| Exon 19 1298 G>A | f | 54 | 6/30 | 6/36 | N/A | N/A | N/A |
| Exon 27 2708delTTAG | f | 56 | 6/60 | 6/60 | Centro-cecal scotoma with constricted fields | Bitemp pallor & pseudocupped; RE 0.5, LE 0.6 | RE TES 908; p 15.05; d 11.92; t 15.08; LE TES 1064; p 13.55; d 11.92; t 11.67 |
| Exon 9 & 11 937AG>TA and 1126 A>G | m | 60 | 6/36 | 6/36 | N/A | N/A | N/A |
| **Normal Vision – NV** | | | | | | |  |
| Deletion of OPA1 | m | 10 | 6/5 | 6/5 | N/A | N/A | Asymp |
| Deletion of OPA1 | m | 11 | 6/9 | 6/9 | N/A | N/A | Asymp |
| Exon 9 & 11 937AG>TA and 1126 A>G | m | 27 | 6/7.5 | 6/7.5 | N/A | N/A | N/A |
| Exon 19 1298 G>A | f | 32 | 6/15 | 6/9 | N/A | N/A | N/A |
| Exon 27 2708delTTAG | f | 37 | 6/6 | 6/6 | NAD | NAD; RE 0.2 LE 0.2 | RE TES 80 TPES 42; LE TES 26 TPES 18 |
| Deletion of OPA1 | f | 46 | 6/6 | 6/6 | N/A | N/A | Asymp |
| Exon 27 2708delTTAG | m | 66 | 6/6 | 6/4.8 | NAD | No pallor | RE TES 196 TPES 117; LE TES 300 TPES 200 |
| **Control – C** | | | | | | |  |
| CEPH 1358 | M | - |  |  |  |  |  |
| CEPH 1444 | M | - |  |  |  |  |  |
| CEPH 1447 | M | - |  |  |  |  |  |
| CEPH 1454 | M | - |  |  |  |  |  |
| CEPH 1459 | M | - |  |  |  |  |  |
| CEPH 1463 | M | - |  |  |  |  |  |
| Non-mutant family member | M | 25 | 6/5. | 6/5. | 1/B 1/B |  | 50s |
| CEPH 1350 | F | 37 |  |  |  |  |  |
| CEPH 1334 | F | 38 |  |  |  |  |  |
| CEPH 1416 | M | 39 |  |  |  |  |  |
| CEPH 1347 | F | 41 |  |  |  |  |  |
| CEPH 1334 | M | 43 |  |  |  |  |  |
| CEPH 1340 | F | 45 |  |  |  |  |  |
| CEPH 1345 | F | 45 |  |  |  |  |  |
| CEPH 1346 | M | 47 |  |  |  |  |  |
| CEPH 1362 | F | 49 |  |  |  |  |  |
| CEPH 1420 | F | 55 |  |  |  |  |  |
| CEPH 1350 | F | 58 |  |  |  |  |  |
| CEPH 1420 | M | 60 |  |  |  |  |  |
| CEPH 1340 | M | 63 |  |  |  |  |  |

**NAD = no abnormality detected, RE = right eye, LE = left eye, N/A = Not available, TES = total error score, TPES = partial error scores**

**Table S1: Table of patient characteristics**

Patients were categorised into the following groups: 1) “normal vision ADOA” where visual acuity was greater than 6/9, 2) “poor vision ADOA” where visual acuity was less than 6/36, or 3) non mutation carrying family member controls or from the well described pedigrees from the HAPMAP population at the Corriell cell repositories (The International HapMap Project: Nature). Patient characteristics including OPA1 mutation and visual acuity for both the left and right eye are shown (CF = count fingers).
