# Supplementary material for: Mitochondrial Oxidative Phosphorylation Compensation May Preserve Vision in Patients with OPA1-Linked Autosomal Dominant Optic Atrophy
Source: PLoS One. 2011 Jun 22;6(6):e21347. doi: 10.1371/journal.pone.0021347 (PMC3120866; doi:10.1371/journal.pone.0021347)
Supplement: Table S2 — Primer pairs used to produce PCR probes spanning entire human mtDNA genome. Primers were designed for the six generated PCR fragments to cover the entire mtDNA genome. Numbers in brackets indicates position alignment to human mtDNA genome. (DOC) [file pone.0021347.s004.doc]

| *Forward Primer* | *Reverse Primer* | *Fragment* |
| --- | --- | --- |
| L3160 (3160-3179) | C1R1 (6506-6528) | A |
| L644 (6441-6463) | H10183 (10164-10183) | B |
| L962 (9622-9646) | ND5H (13177-13200) | C |
| G1F1 (13145-13168) | A1R2 (16514-16540) | D |
| HUB13 (15749-15769) | H330 (3272-3306) | E |

**Table S2: Primer pairs used to produce PCR probes spanning entire human mtDNA genome.**

Primers were designed for the six generated PCR fragments to cover the entire mtDNA genome. Numbers in brackets indicates position alignment to human mtDNA genome.
