# Supplementary material for: Mitochondrial Oxidative Phosphorylation Compensation May Preserve Vision in Patients with OPA1-Linked Autosomal Dominant Optic Atrophy
Source: PLoS One. 2011 Jun 22;6(6):e21347. doi: 10.1371/journal.pone.0021347 (PMC3120866; doi:10.1371/journal.pone.0021347)
Supplement: Table S3 — Antibodies used for western blotting. A list of commercially available antibodies that were used for western blotting. (DOC) [file pone.0021347.s005.doc]

| **Primary** | **Supplier** | **Dilution of primary antibody** |
| --- | --- | --- |
| Complex IV, subunit II | Invitrogen | 1:5000 |
| Complex IV, subunit Va | Invitrogen | 1:5000 |
| Actin | Sigma | 1:10000 |
| Porin | Abcam | 1:5000 |
| oxidative phosphorylation cocktail MS 604 (Complex I subunit NDUFB8, Complex II subunit 30kDa, Complex III subunit Core 2, Complex IV subunit I, ATP synthase subunit alpha) | Mitosciences | 1:500 |
| Opa1 | BD Biosciences | 1:10000 |
| mtTFAM | Santa Cruz | 1:1000 |
| NRF1 | Abcam | 1:1000 |

**Table S3: Antibodies used for western blotting**
